# Outcomes of pregnancy in Wilson’s disease: a population-based study from multiple centres of the Han population in China

**DOI:** 10.3389/fmed.2024.1436828

**Published:** 2024-08-23

**Authors:** Rao Rao, Xu-En Yu, Zhi-Hua Zhou, Shan Shu, Yi-Gang Du, Yong-Zhu Han, Yong-Sheng Han

**Affiliations:** ^1^Institute of Neurology, Anhui University of Chinese Medicine, Hefei, China; ^2^Department of Neurology, Affiliated Hospital of Neurology Research Institute of Anhui University of Chinese Medicine, Hefei, China; ^3^Department of Neurology, The First Affiliated Hospital of Guangdong Pharmaceutical University, Guangzhou, China; ^4^Department of Neurology, Anhui Provincial Hospital of Integrated Traditional Chinese and Western Medicine, Hefei, China; ^5^Wannan Medical College, Wuhu, China; ^6^Center for Xin’an Medicine and Modernization of Traditional Chinese Medicine of IHM, Anhui University of Chinese Medicine, Hefei, China

**Keywords:** Wilson’s disease, outcome of pregnancy, retrospective study, prediction, urinary copper

## Abstract

**Objectives:**

Wilson’s disease is an autosomal recessive disorder related to copper metabolism which mostly patients occurs in adolescents, fertility has become a problem that WD needs to face.

**Methods:**

A 21 years retrospective follow up study was conducted and a total of 220 female patients were included to identify patients with outcomes of pregnancy.

**Results:**

Untreated female patients with WD had a spontaneous abortion rate of 44%. During the study period, 146 female patients with WD from multicenter, 75 patients (51.4%) had successful outcomes of pregnancy. Notably, urinary copper levels below 616 μg/24 h were strongly associated with successful pregnancy. The nomogram built on these variables were age, urinary copper, haemoglobin and Child–Pugh classification, internally validated and showed good performance.

**Conclusion:**

The spontaneous abortion rate was 44% in untreated females with WD and developed a four-variable risk prediction model to accurately predict the likelihood of a successful pregnancy.

## 1 Introduction

Wilson’s disease (WD) is an autosomal recessive disorder related to copper metabolism resulting from mutations in the ATP7B gene. It has a global incidence ranging from 1:29,000 to 40,000 individuals (1–3). The condition is characterised by the deposition of excess copper ions in various organs, predominantly in the liver and brain, leading to clinical presentations of liver damage and neurological/psychiatric symptoms (4, 5).

While WD affects approximately 46% of diagnosed cases involve females, and mostly patients occurs in adolescents, fertility has become a problem that WD needs to face. Fertility issues in women of childbearing age with WD are often easily overlooked, and there exists a scarcity of information regarding pregnancy outcomes in these patients (6). Existing literature suggests a high miscarriage (or recurrent miscarriages) rate in untreated female patients with WD, some patients even occurs with recurrent miscarriage (7). Consequently, managing pregnancies in women with WD stands as an important clinical issue. This study aims to enhance pregnancy management, improve successful pregnancy outcomes and prevent exacerbation of post-pregnancy symptoms.

## 2 Materials and methods

### 2.1 Patients

We retrospectively analysed the fertility of female patients with WD from January 2000 to December 2021 which conducted using a combination of questionnaires and telephone. The patients had received no treatment before pregnancy. Patients with infertility unrelated to WD and those receiving gynaecological treatment were excluded.

Given the elevated rate of patient miscarriage, there is an urgent imperative for a clinical predictive model to prognosticate pregnancy outcomes in women with WD.

**Figure 1:**
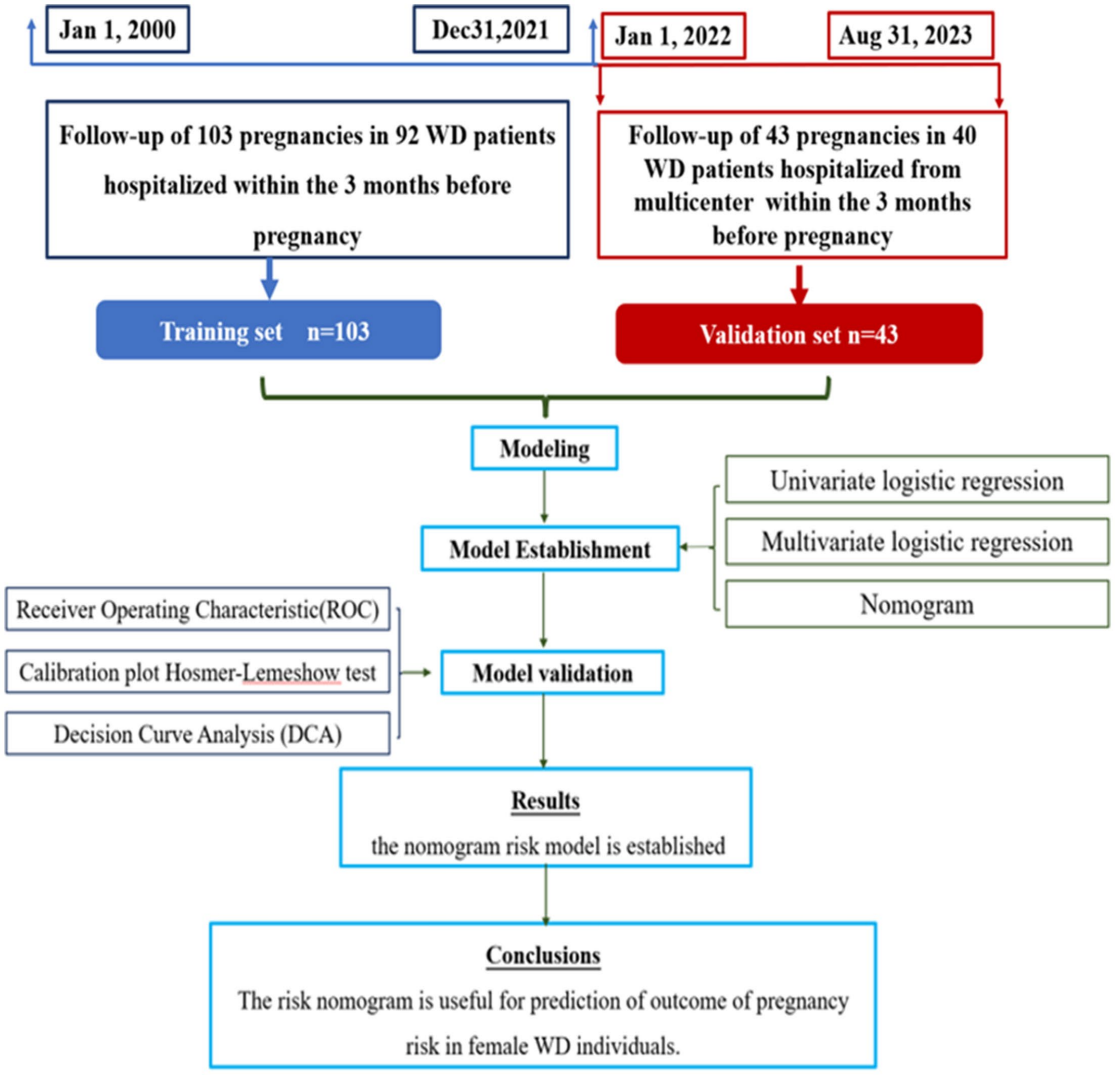
Flow diagram.

The predictive model was derived from the analysis of 103 medical records of 92 patients who sought care from multicenter (Affiliated Hospital of Neurology Research Institute of Anhui University of Chinese Medicine, Anhui Provincial Hospital of Integrated Traditional Chinese and Western Medicine and the First Affiliated Hospital of Guangdong Pharmaceutical University) within 3 months before pregnancies, spanning from January 2000 to December 2021. Follow-up assessments clarified the pregnancy outcomes. For validation cohort, a separate set of patients who conceived within 3 months of treatment at these hospitals, between January 2022 to October 2022 were selected. The cases for successful pregnancies and abortions were selected in approximately equal numbers for the purpose of statistical analysis (Figure 1).

### 2.2 Variables of the study

The variable among outcomes of pregnancy in Wilson’s disease (yes/no). Prognostic determinants were: age at conception, spouse’s age at conception, age of onset, duration of disease, ceruloplasmin levels, serum copper, copper oxidase, urinary copper, white blood cell count (WBC), red blood cell count (RBC), haemoglobin (HB), platelet (PLT), total bilirubin (TBIL), direct bilirubin (DBIL), indirect bilirubin (IBIL), albumin (ALB), globulin (GLB), glutamic oxalacetic transaminase (ALT), glutamic oxalacetic transaminase (AST), acetylcholine (AECH), urea nitrogen (BUN), creatinine (Cr) and uric acid (UA). Additionally, Child–Pugh classification, Kayser-Fleischer Ring presence and UWDRS-I (Part-1 of United Wilson’s Disease Rating Scale) were documented.

### 2.3 Operational definitions

#### 2.3.1 Age at conception

Refers to the specific age of a woman at the time she conceives a child. This crucial factor plays a significant role in various aspects of reproductive health and pregnancy outcomes.

#### 2.3.2 Spouse’s age at conception

The spouse’s age at the time of pregnancy. Spouse’s age at conception plays a significant role in determining various aspects of the child’s development and health.

#### 2.3.3 Urinary copper

All of patients with copper chelation therapy. This manuscript specifically refers to the last recorded copper urine before pregnancy with sufficient chelating agent usage. The disorder of copper metabolism known as WD can be assessed by measuring urinary copper levels, which serve as a crucial indicator for evaluating the body’s copper status.

#### 2.3.4 Child–Pugh classification

The “Child–Pugh classification” is a widely used scoring system in medicine that assesses the severity of liver disease and predicts patient prognosis, plays a crucial role in evaluating the severity of liver disease and guiding clinical management decisions.

#### 2.3.5 Kayser-Fleischer Ring

The “Kayser-Fleischer Ring” is a medical term typically seen in the cornea of the eye, appears as a brownish or greenish discoloration encircling the iris. The presence of Kayser-Fleischer rings is closely associated with Wilson’s disease.

#### 2.3.6 UWDRS-I (Part-1 of United Wilson’s Disease Rating Scale, neurological subscale)

UWDRS-I is an essential component in assessing the severity and progression of neurological Wilson’s disease, plays a crucial role in comprehensively evaluating the neurological symptoms associated with Wilson’s disease.

### 2.4 Statistical methods

R software R Core Team (8) is utilized in this study. The derivation cohort is employed for model training, while the validation cohort is used for model verification. Continuous variables were presented as the mean and standard deviation or medians and interquartile ranges, as appropriate. Categorical variables were presented as numbers and percentages. Categorical variables were compared using chi-square test.

Based on the training cohort, the steps for constructing a predictive model are as follows: firstly, potential predictors of the outcome event are identified using single-factor logistic regression analysis (*p* < 0.05). For these selected variables, multiple-factor logistic regression analysis is conducted using stepwise (bidirectional) method. A column line chart is constructed based on variables with *p* < 0.05 from the stepwise method in order to build a predictive model. The constructed column line chart undergoes bootstrap sampling validation 1,000 times to draw a calibration curve for assessing model calibration and an HL test for evaluating model goodness-of-fit. Furthermore, receiver operating characteristic (ROC) curve analysis is performed to calculate metrics such as area under the curve (AUC), sensitivity, specificity, in order to evaluate its discriminatory performance. Clinical decision curves (DCA) are created to assess the clinical utility of the model by quantifying net benefits within threshold probability ranges. Finally, the constructed model is validated in a validation cohort.

The statistical analysis was performed using R software (version 4.3.2). Statistical significance was determined by a two-sided test with a *p*-value threshold of <0.05.

## 3 Results

### 3.1 Clinical characteristics and outcome of pregnancy in 220 untreated female patients

**Figure 2:**
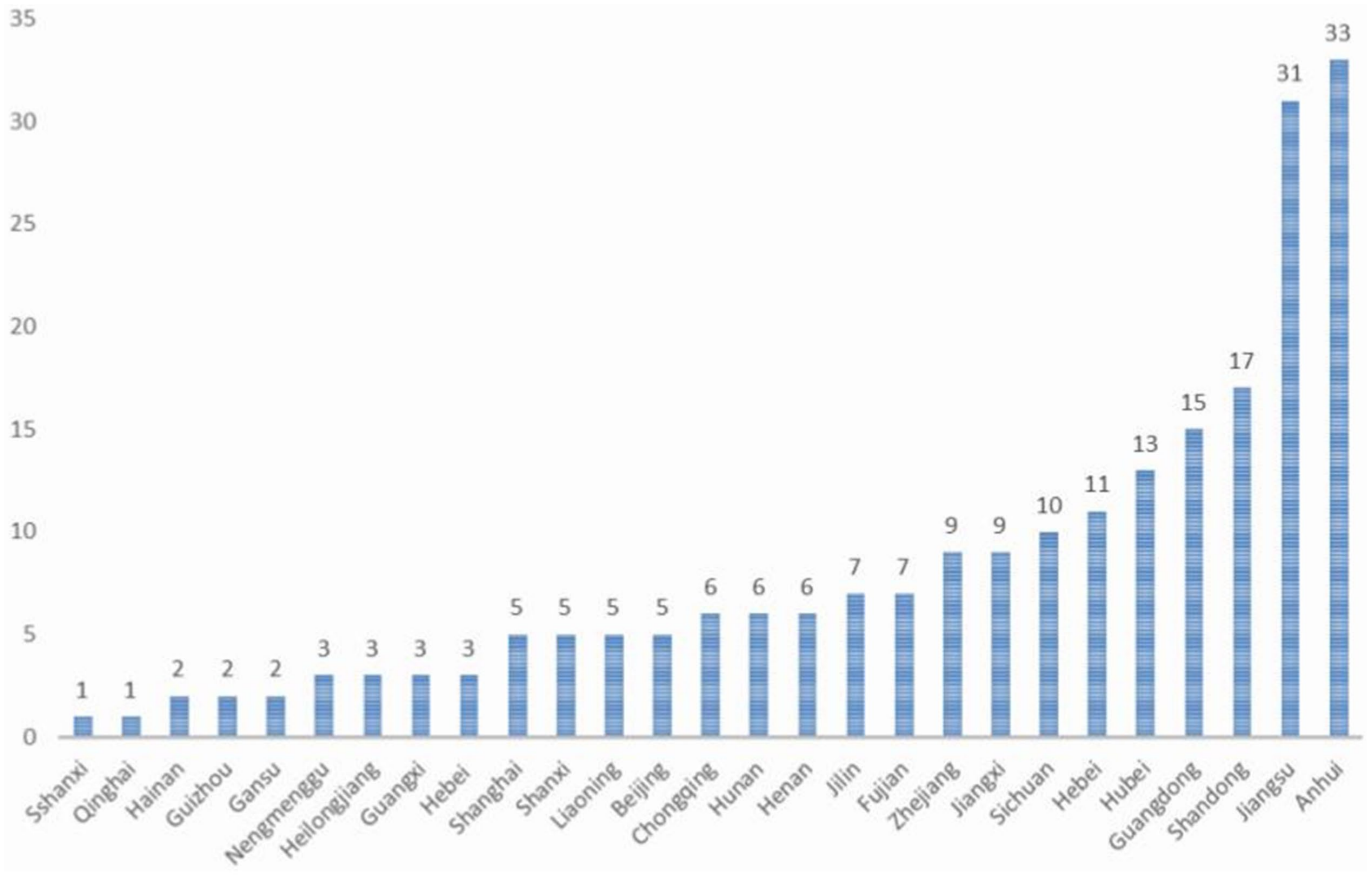
Distribution of 220 patients by province.

**Figure 3:**
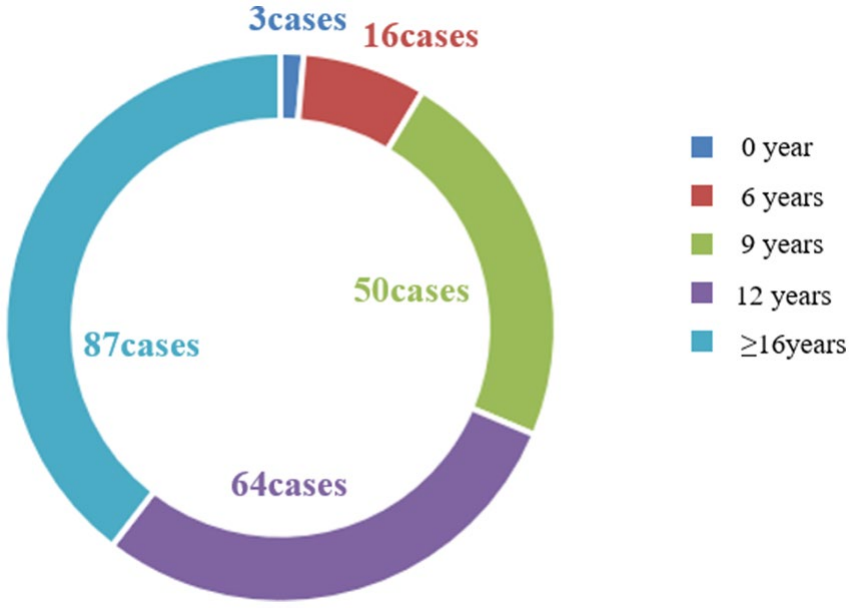
Composition ratio of 220 patients’ years of education.

This study encompassed an observation of 499 pregnancies in 220 female patients with WD, constituting the most extensive clinical dataset available in the current literature. These 220 women with WD hailed from 27 provinces, cities, and autonomous regions in China (Figure 2), all of Han nationality. The majority, accounting for 39.54% of the total, had received 16 or more years of education, while approximately 29.09% had 12 years of education (Figure 3), which is higher than that of the general population.

Among these patients, five female patients with WD experienced three successful pregnancies, while two patients delivered twins, accounting for 4% of the total, a proportion similar to that of the healthy population (9). Within the 499 pregnancies, there were 268 miscarriages, excluding 48 abortions for non-foetal developmental reasons, resulting in a 44% spontaneous miscarriage rate (220/499), which is consistent with that in the literature (6) and higher than that in normal Chinese females (10). The most prevalent causes of 220 spontaneous abortions were stillbirth and fetal dysplasia/malformation.

Overall, the 220 females with WD delivered a total of 229 fetuses, with 75 females having no history of abortion. Notably, one patient had the highest number of miscarriages, eight, as due to foetal development cessation in the third month of pregnancy, with a subsequent diagnosis of WD following recurrent miscarriages, similar to a case reported in the literature (6). Unfortunately, this patient had already passed the childbearing age at the time of diagnosis, remaining infertile thereafter. Additionally, one patient delivered a fetus diagnosed with WD.

### 3.2 Model development and validation

#### 3.2.1 Predictor selection

Baseline features of study population by female patients with WD and Characteristics of patients with WD in derivation and validation cohorts were performed (Tables 1, 2).

**Table 1:** Baseline features of study population by female patients with WD in derivation cohor.

Variables	Abortion	Successful outcomes of pregnancy	*p*
	71	75	
Age (years; median; IQR)	31.00 (28.00, 34.00)	28.00 (24.00, 32.00)	0.002
Spouse’s age (years; $\bar{x}\;\pm \;s$ )	32.23 ± 4.74	29.77 ± 5.32	0.004
Age of onset (years; median; IQR)	4.00 (4.00, 5.00)	4.00 (3.00, 5.00)	0.119
Duration of disease (years; median; IQR)	16.00 (13.00, 21.00)	14.00 (12.00, 20.50)	0.219
Cp (mg/L; median; IQR)	61.50 (43.85, 78.50)	55.00 (46.15, 75.25)	0.833
Cu (μmol/L; median; IQR)	3.83 (2.29, 5.46)	4.32 (3.04, 5.85)	0.156
Sco (OD; median; IQR)	0.04 (0.03, 0.05)	0.04 (0.03, 0.05)	0.845
Urinary copper (μg/24 h; median; IQR)	943. 61 (797.90, 1138.32)	752.52 (682.84, 851.51)	<0.001
WBC (×10^9^/L; median; IQR)	4.50 (3.35, 5.70)	4.50 (3.45, 5.55)	0.938
RBC (×10^12^/L; median; IQR)	4.20 (3.73, 4.46)	4.38 (4.05, 4.61)	0.017
HGB (g/L; median; IQR)	121.00 (108.00, 128.00)	126.00 (116.00, 132.00)	0.016
PLT (×10^9^/L; median; IQR)	105.00 (73.50, 147.50)	106.00 (75.50, 134.00)	0.608
TBIL (μmol/L; median; IQR)	14.60 (11.35, 22.45)	15.20 (11.50, 23.20)	0.950
DBIL (μmol/L median; IQR)	4.50 (2.80, 7.00)	4.50 (2.65, 6.50)	0.986
IBIL (μmol/L; median; IQR)	9.80 (7.90, 14.45)	10.30 (7.30, 13.85)	0.776
ALB (g/L; median; IQR)	40.80 (37.05, 44.85)	43.10 (38.75, 45.60)	0.089
GLB (g/L; median; IQR)	25.30 (21.10, 29.40)	25.40 (22.30, 28.75)	0.534
ALT (U/L; median; IQR)	28.00 (18.00, 44.80)	22.00 (15.50, 38.50)	0.065
AST (U/L; median; IQR)	29.00 (23.00, 38.50)	28.00 (20.50, 35.00)	0.084
ACHE (U/L; $\bar{x}\;\pm \;s$ )	5144.27 ± 2042.85	5338.05 ± 1676.55	0.533
BUN (mmol/L; median; IQR)	5.18 (4.16, 6.14)	5.12 (4.16, 6.13)	0.900
Cr (mmol/L; median; IQR)	55.00 (45.00, 65.00)	60.00 (50.50, 73.50)	0.105
UA (mmol/L; median; IQR)	129.00 (86.00, 165.50)	105.00 (78.00, 143.50)	0.190
Child–Pugh			0.005
A	39 (54.9)	54 (72)	
B	15 (21.1)	17 (22.7)	
C	17 (23.9)	4 (5.3)	
Kayser-Fleischer Ring			0.070
Existence	31 (43.7)	44 (58.7)	
Absence	40 (56.3)	31 (41.3)	
UWDRS-I (median; IQR)	28.00 (15.00, 71.50)	44.00 (16.00, 86.00)	0.065

**Table 2:** Characteristics of patients with WD in derivation and validation cohorts.

Variables	Derivation	Validation	*p*
*N*	103	43	
Outcomes of pregnancy (%)	53 (51%)	22 (51%)	1.000
Age (years; median; IQR)	29.00 (25.00–33.00)	30.00 (28.00–34.00)	0.071
Spouse’s age (years; $\bar{x}\;\pm \;s$ )	30.28 ± 5.27	32.60 ± 4.57	0.120
Age of onset (years; median; IQR)	15 (12–21)	18 (11–21)	0.841
Duration of disease (years; median; IQR)	12 (10–16)	14 (8–19)	0.569
Cp (mg/L; median; IQR)	56.40 (44.30–76.20)	57.50 (44.10–76.10)	0.862
Cu (μmol/L; median; IQR)	4.62 (3.00–5.65)	3.40 (2.30–5.10)	0.024
Sco (OD; median; IQR)	0.04 (0.03–0.05)	0.03 (0.02–0.066)	0.744
Urinary copper (μg/24 h; median; IQR)	818.69 (693.06–936.21)	871.20 (765.75–1151.04)	0.003
WBC (×10^9^/L; median; IQR)	4.50 (3.30–5.50)	4.80 (3.50–5.70)	0.447
RBC (×10^12^/L; median; IQR)	4.30 (3.87–4.61)	4.25 (3.76–4.40)	0.144
HGB (g/L; median; IQR)	126 (111–131)	121 (113–130)	0.255
PLT (×10^9^/L; median; IQR)	104.0 (75.0–138.0)	111.0 (69.0–168.0)	0.485
TBIL (μmol/L; median; IQR)	14.90 (11.50–22.40)	14.70 (11.10–24.70)	0.817
DBIL (μmol/L median; IQR)	4.50 (2.70–6.60)	4.50 (2.60–6.70)	0.955
IBIL (μmol/L; median; IQR)	10.10 (7.50–13.70)	10.20 (7.10–16.70)	0.529
ALB (g/L; median; IQR)	42.1 (38.4–45.8)	42.1 (37.4–44.9)	0.363
GLB (g/L; median; IQR)	24.0 (20.6–28.0)	27.6 (24.9–31.9)	<0.01
ALT (U/L; median; IQR)	26.0 (17.0–44.2)	20.0 (14.0–34.0)	0.022
AST (U/L; median; IQR)	29.0 (22.0–38.0)	28.0 (21.00–38.00)	0.817
ACHE (U/L; $\bar{x}\;\pm \;s$ )	5206.3 ± 1772.52	5334.33 ± 2073.25	0.163
BUN (mmol/L; median; IQR)	5.25 (4.38–6.48)	4.81 (3.77–5.87)	0.052
Cr (mmol/L; median; IQR)	60.0 (51.0–76.0)	51.0 (41.0–61.0)	<0.01
UA (mmol/L; median; IQR)	107 (82–152)	134 (88–174)	0.477
Child–Pugh			0.243
A	64	29	
B	21	11	
C	18	3	
K-F Ring			0.720
Existence	54	21	
Absence	49	22	
UWDRS-I (median; IQR)	28 (15–76)	44 (24–76)	0.117

After review of literatures, 27 predictors demographic and birth characteristics, newborn interventions and diagnosed clinical comorbidities, baseline laboratory profiles of the neonate and maternal obstetric were considered to predict outcome of pregnancy.

Major one-way logistic regression of independent prognostic factors identified age at pregnancy preparation, spouse’s age at preparation, last urinary copper levels following adequate intravenous copper chelation before conception, Child–Pugh classification, RBC, haemoglobin and cholinesterase levels were significantly correlated with pregnancy success probability.

Subsequent, multivariate analysis confirmed the significance of age at pregnancy preparation, last urinary copper levels following adequate intravenous copper chelation before conception, haemoglobin and Child–Pugh classification in determining pregnancy success. Urinary copper was identified as an independent risk factor for the probability of pregnancy success, while the spouse’s age, RBC count and cholinesterase were ultimately excluded from the final model (Table 3).

**Table 3:** Univariate logistic regression and multivariate logistic regression in the derivation cohort for patients with WD.

	Univariate logistic regression		Multivariate logistic regression	
	OR (95% CI)	*p*	OR (95% CI)	*p*
Age	0.91 (0.84–0.99)	0.023	0.86 (0.78–0.96)	0.006
Spouse’s age	0.91 (0.84–0.98)	0.019		
Age of onset	0.96 (0.89–1.04)	0.320		
Duration of disease	0.95 (0.89–1.02)	0.183		
Child–Pugh				
A	Reference		Reference	
B	0.75 (0.28, 2.05)	0.574	0.65 (0.20, 2.08)	0.459
C	0.20 (0.05, 0.61)	0.009	0.22 (0.05, 0.84)	0.033
Cp	1.00 (0.90–1.01)	0.944		
Cu	1.11 (0–5146270.98)	0.358		
Sco	0.77 (0.00–2637.42)	0.974		
Urinary copper	1 (0.99–1)	<0.01	0.99 (0.99–1)	<0.001
WBC	1.1 (0.86–1.4)	0.465		
RBC	2.84 (1.36–5.93)	0.006		
HGB	1.04 (1.00–1.07)	0.020	1.05 (1.01–1.09)	0.016
PLT	1 (0.99–1.01)	0.955		
TBIL	0.97 (0.93–1.02)	0.21		
DBIL	0.93 (0.87–1.01)	0.068		
IBIL	1.00 (0.94–1.07)	0.963		
ALB	1.04 (0.98–1.11)	0.167		
GLB	1.05 (1.00–1.11)	0.056		
ALT	0.98 (0.97–1.00)	0.069		
AST	0.98 (0.95–1.01)	0.148		
ACHE	1.000 (1.000–1.000)	0.03		
BUN	0.93 (0.78–1.10)	0.395		
Cr	1.01 (0.99–1.03)	0.311		
UA	1.00 (0.99–1.00)	0.525		
K-F Ring				
Existence	Reference			
Absence	0.605 (0.275, 1.313)	0.206		
UWDRS-1	1.01 (1–1.02)	0.134		

#### 3.2.2 Independent prognostic factors for prediction of successful pregnancy in female patients with WD

The strongest correlation was observed with urinary copper (this manuscript specifically refers to the last recorded copper urine before pregnancy with sufficient chelating agent usage), with a cut-off value at 661 μg/24 h in derivation cohort, 616 μg/24 h in validation cohort, suggesting a higher likelihood of successful pregnancies with urinary copper below this threshold (Table 4).

**Table 4:** Prediction efficiency of Urinary copper in the derivation cohort and validation cohorts for patients with WD.

	Derivation cohort	Validation cohorts
Cutoff (mg/24 h)	0.661	0.616
AUC	0.828 (0.748, 0.907)	0.857 (0.740, 0.974)
*p*-value	<0.001	<0.001
Sensitivity	0.660 (0.533, 0.788)	0.773 (0.598, 0.948)
Specificity	0.900 (0.817, 0.983)	0.857 (0.707, 1.000)
Accuracy	0.777 (0.773, 0.780)	0.814 (0.807, 0.821)
Positive predictive value	0.875 (0.773, 0.977)	0.850 (0.694, 1.006)
Negative predictive value	0.714 (0.603, 0.826)	0.783 (0.614, 0.951)
KAPPA	0.556 (0.401, 0.711)	0.629 (0.397, 0.860)
Youden index	0.560	0.630
HL test	*p* = 0.373	*p* = 0.840
Cindex_boot1000	0.828 (0.743 to 0.898)	0.857 (0.719 to 0.952)

#### 3.2.3 Nomogram for prediction of successful pregnancy in female patients with WD

**Figure 4:**
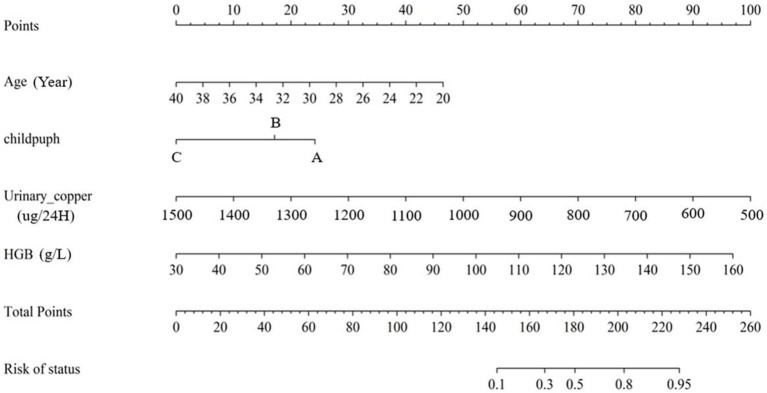
Risk prediction nomogram for successful pregnancy in female patient with WD.

The risk prediction graph for successful pregnancies in female patients with WD was constructed based on four significant independent factors observed in the derived cohort: age at pregnancy preparation, urinary copper, haemoglobin, Child–Pugh classification. Each patient received a score indicating the probability of eventual pregnancy success (Figure 4).

**Figure 5:**
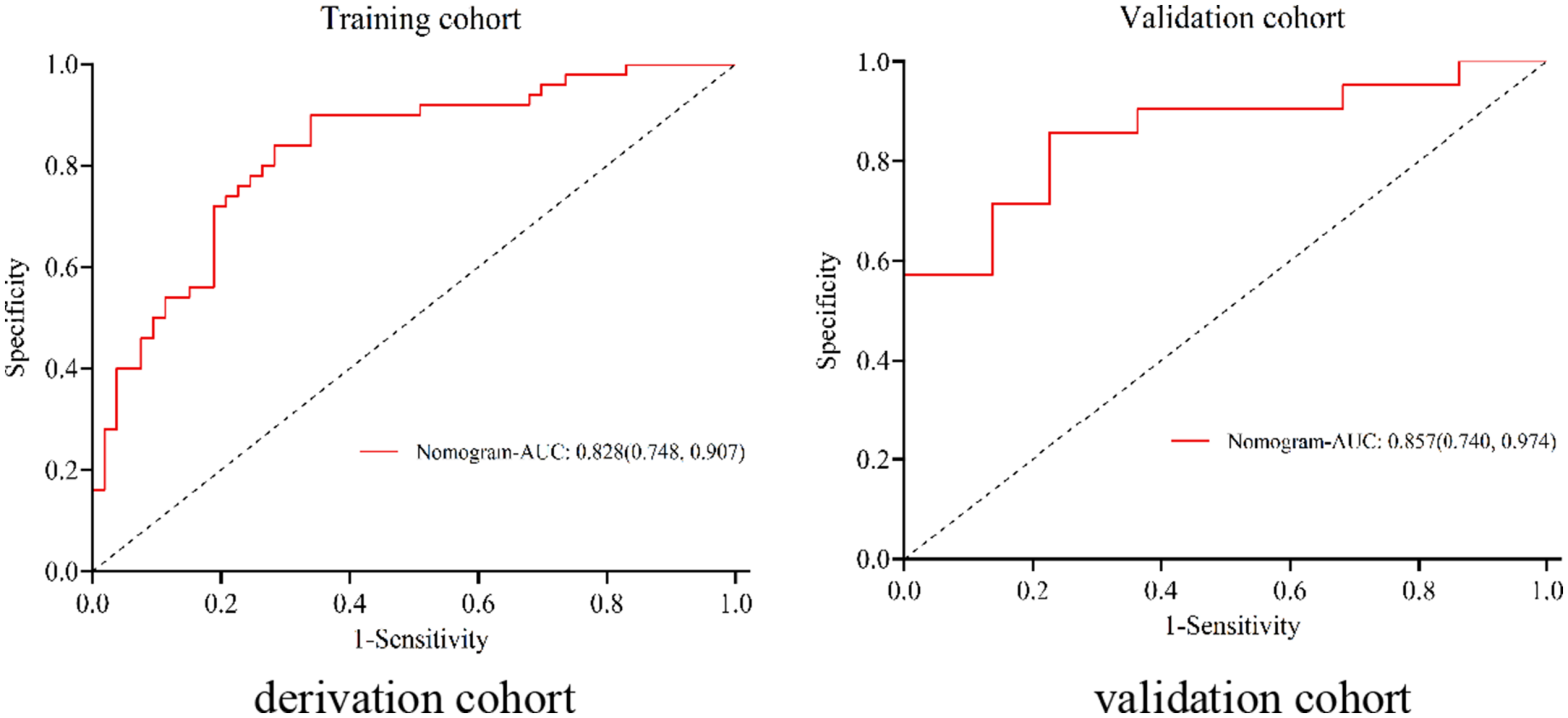
ROC in the derivation cohort and validation cohort for patients with WD.

**Figure 6:**
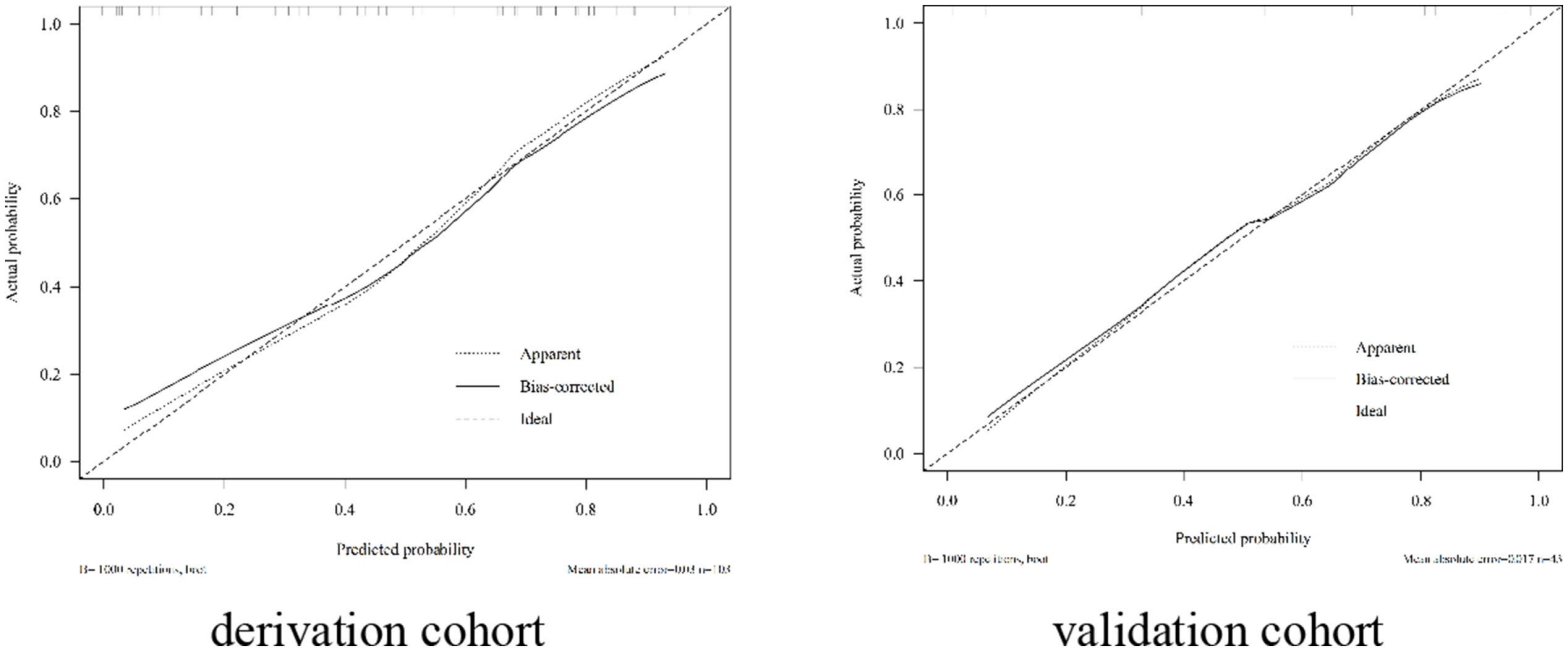
Calibration plot in the derivation cohort and validation cohort for patients with WD.

**Figure 7:**
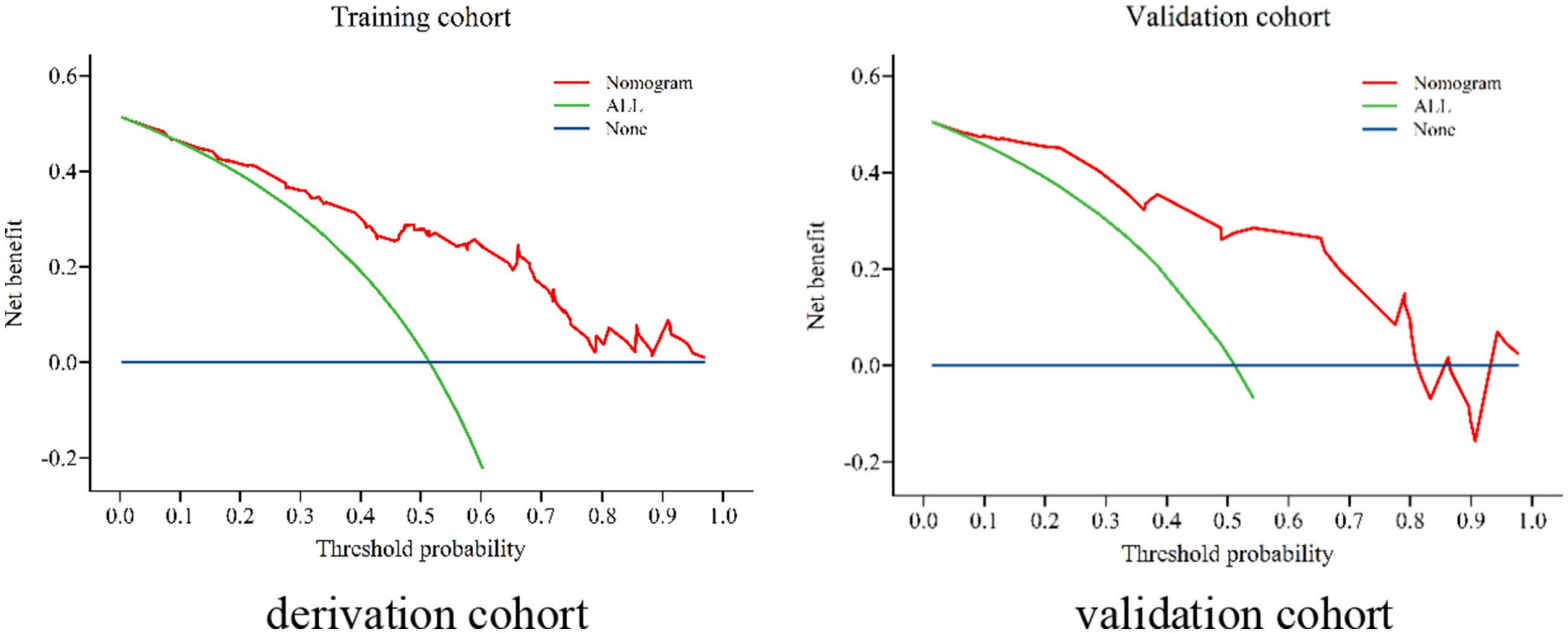
Decision curve analysis (DCA) in the derivation cohort and validation cohort for patients with WD.

The nomogram exhibited significant differential power with an AUC of 0.828 (95% CI 0.748–0.907) for predicting pregnancy success. Calibration plots of the probability of pregnancy success indicated consistent predictions with actual observations. The nomogram demonstrated robust discriminatory ability with an AUC value of 0.857 (95% CI 0.740–0.974) and showed good agreement between predicted and observed outcomes in successful pregnancy occurrences (Figure 5). Evaluate goodness of fit in Hosmer–Lemeshow test of derivation cohorts (Chi-square 5.977, *p*-value 0.742) and validation cohorts (Chi-square 13.774, *p*-value 0.131) (see Figure 6). Moreover, there was no significant difference in the AUC values between the derivation and validation cohorts (Figure 7).

## 4 Discussion

The onset of WD typically occurs between the ages of 5 and 35, most often in adolescents, WD clinical symptoms vary widely from asymptomatic disease to acute liver failure or chronic liver disease with or without neuropsychiatric symptoms. Clinical indicators demonstrate a gender effect, with hepatic manifestations more common in female patients with WD (11). Fertility is a significant concern for women of childbearing age as opposed to men.

Pregnancies in individuals with WD have been associated with altered copper metabolism (12), similar to the effects of copper-containing intrauterine devices that produce contraception by generating excess free copper ions. Similarly, high plasma concentrations of free intrauterine copper could be the cause of miscarriages that are common in some untreated WD cases (13). Additionally, copper accumulation in the endometrium might impact embryo implantation, leading to failed embryo transfers (14). During pregnancy, high copper levels have been associated with intrauterine growth restriction (15).

Fertility studies first reported on WD can be traced back to 1959 (7). Since then, the issue of fertility in women with WD has gradually gained attention.

Untreated WD in women of childbearing age presents with decreased menstruation or amenorrhea, conception difficulties, recurrent miscarriages and complications such as preeclampsia and placental abruption. Untreated WD has also been reported to result in low fertility, even when pregnancy occurs, often leading to spontaneous abortion (16–20). The risk of spontaneous abortion is one of the most common pregnancy complications in obstetrics and gynaecology. The risk of one-time spontaneous miscarriage in Chinese women of reproductive age is estimated to be 10%, and the incidence of recurrent miscarriage is between 1 and 5% (10). A 2018 retrospective multicentre study of 282 pregnancies in 136 patients with WD reported that the rate of miscarriage in patients with untreated WD was as high as 40% (7), with several small-sample studies suggesting a rate of 20–50% (13). Treated WD patients also demonstrate a higher risk of spontaneous abortion compared to controls with or without liver disease (21). In this Chinese cohort of WD pregnancies, the untreated female patients experienced a 44% spontaneous abortion rate and were prone to recurrent abortions.

The high rate of abortion in patients with WD, if left untreated, poses serious economic burdens on the patient and their families and also on their physical and mental well-being. The previous study conducted at our center reported a total of 117 pregnancies in 75 cases of patients with Wilson’s disease (WD). Among these, 108 pregnancies were successful while nine resulted in spontaneous abortions, indicating a relatively low spontaneous abortion rate of 7.7% (22). These findings suggest that women with WD can safely pursue pregnancy after anti-copper therapy.

This study was aimed to develop a prediction tool (nomogram) that helps to predict outcome of pregnancy in female WD patients. Prediction models can help health professionals to make clinical decisions through patient risk stratification with the hope of improving patient outcomes of pregnancy and quality of life.

Successful pregnancy was correlated with the age of the female patient with WD at pregnancy preparation, the age of the spouse at pregnancy preparation, urinary copper, haemoglobin, RBC, cholinesterase levels and the Child–Pugh classification.

WD is a rare genetic disorder that affects the body’s ability to metabolize copper properly. The excessive accumulation of copper in the uterus and ovaries can have significant implications for fertility. When copper levels become elevated in these reproductive organs, it can disrupt normal hormonal balance and interfere with crucial processes involved in conception and pregnancy. As a result, individuals with Wilson’s disease may experience difficulties conceiving or maintaining a healthy pregnancy.

To address this issue effectively, it becomes essential to reduce the overall burden of copper within the body. Fertility studies first reported on WD which had a successful pregnancy after anti-copper therapy, with a well-developed fetus (7). Monitoring urinary copper levels serves as a vital indicator for evaluating the extent of copper accumulation within the system.

In clinical practice, the monitoring of urinary copper levels plays a crucial role in the assessment and diagnosis of WD. By quantifying the concentration of copper in urine samples, healthcare professionals can obtain valuable insights into an individual’s overall copper metabolism. Urinary copper was a key indicator, emphasising the importance of achieving levels below 616 μg/24 h for a safer pregnancy.

Pregnancy is a unique clinical state marked by several normal physiological changes affecting various body organs including the liver (23). It is prudent for patients with WD, especially when liver damage is severe, to manage pregnancy well to improve the outcome. Maintaining treatment during pregnancy is vital to prevent severe disease regression (24). The severity of liver damage is closely associated with the occurrence of pregnancy in WD patients.

The first successful WD pregnancy in China was reported in 1991. The patient became pregnant after 2 years of d-penicillamine treatment and had a successful pregnancy. However, the use of chelation therapy during pregnancy remains controversial (6). It was once speculated that continuous penicillamine treatment throughout pregnancy could protect the mother from WD recurrence, with no risk to the fetus (25). However, it was not until after 2010 that a consensus was attained, with national and international literature agreeing that copper chelating therapy is important for women with WD in the preparatory period and should subsequently be reduced during pregnancy. The rate of spontaneous abortion was significantly lower in patients who adhered to this treatment compared to those who did not receive treatment, and the rate of spontaneous abortion was significantly lower after penicillamine treatment (7, 26–28). Hence, anti-copper therapy is essential for female WD patients who are planning to conceive.

The best management method for pregnancy in women with WD may be intensive pre-pregnancy copper chelation therapy. Pregnancy is considered a high-risk period necessitating regular monitoring (29). Literature suggests conflicting findings regarding WD symptom progression during pregnancy, which poses various challenges for clinicians. Some literature suggests the observation of clinically significant improvement in the severity of WD during pregnancy and the months following pregnancy (30). However, some reported that the symptoms may worsen. Nevertheless, the management of neuropsychiatric symptoms, hepatic symptoms and renal symptoms during pregnancy is a clinical challenge as the use of medication may harm the fetus, while the lack of medication may lead to the worsening of the patient’s symptoms and also affect the health of the fetus (31, 32). In the present study, intravenous copper repellent combined with the improvement of hepatic and cerebral function before pregnancy preparation was found to significantly improve the pregnancy success rate in patients with a young age, urinary copper (The last recorded copper urine before pregnancy with sufficient chelating agent usage) level less than 616 μg/24 h, normal haemoglobin levels and a Child–Pugh grade of A. Achieving the relevant indicators before pregnancy will significantly increased the success rate of pregnancy, less likely to have worsening symptoms in pregnancy, and the fetus was also safer.

This study suggests that patients should manage their disease well during pregnancy preparation, emphasising a urinary copper level below 616 μg/24 h following sufficient intravenous copper chelation prior to pregnancy preparation. Therefore, the best management method for pregnancy in women with WD could be intensive pre-pregnancy anti-copper therapy.

However, this study also has certain limitations, and we will continue to expand the sample size and collect more indicators in the future, so as to make greater contribution to guiding WD patients’ pregnancy. Due to the large span of time, incomplete collection of many data such as brain magnetic resonance imaging data, and the majority of pregnant WD patients are not hospitalized, this study still needed to confirm in further clinical practice.

## Data Availability

The raw data supporting the conclusions of this article will be made available by the authors, without undue reservation.
